# DNA methylation‐based classification of glioneuronal tumours synergises with histology and radiology to refine accurate molecular stratification

**DOI:** 10.1111/nan.12894

**Published:** 2023-03-08

**Authors:** Thomas J. Stone, Kshitij Mankad, Ai Peng Tan, Wajanat Jan, Jessica C. Pickles, Maria Gogou, Jane Chalker, Iwona Slodkowska, Emily Pang, Mark Kristiansen, Gaganjit K. Madhan, Leysa Forrest, Deborah Hughes, Eleni Koutroumanidou, Talisa Mistry, Olumide Ogunbiyi, Saira W. Ahmed, J. Helen Cross, Mike Hubank, Darren Hargrave, Thomas S. Jacques

**Affiliations:** ^1^ Developmental Biology and Cancer Research and Teaching Department UCL Great Ormond Street Institute of Child Health 30 Guilford Street London WC1N 1EH UK; ^2^ Department of Histopathology Great Ormond Street Hospital for Children NHS Foundation Trust Great Ormond Street London WC1N 3JH UK; ^3^ Department of Radiology Great Ormond Street Hospital for Children NHS Foundation Trust Great Ormond Street London WC1N 3JH UK; ^4^ Department of Diagnostic Radiology National University of Singapore 21 Lower Kent Ridge Road 119077 Singapore; ^5^ A*STAR Research Entities (ARES) Singapore Institute for Clinical Sciences (SICS) Singapore; ^6^ Department of Imaging Imperial College Healthcare NHS Trust London UK; ^7^ Developmental Neurosciences Research and Teaching Department UCL Great Ormond Street Institute of Child Health 30 Guilford Street London WC1N 1EH UK; ^8^ Specialist Integrated Haematology and Malignancy Diagnostic Service Great Ormond Street Hospital for Children NHS Foundation Trust Great Ormond Street London WC1N 3JH UK; ^9^ UCL Genomics Zayed Centre for Research into Rare Disease in Children 20 Guilford Street London WC1N 1DZ UK; ^10^ Centre for Molecular Pathology Royal Marsden Hospital London SM2 5NG UK; ^11^ Department of Haematology and Oncology Great Ormond Street Hospital for Children NHS Foundation Trust Great Ormond Street London WC1N 3JH UK

**Keywords:** dysembryoplastic neuroepithelial tumour, ganglioglioma, glioneuronal tumour, machine learning, molecular pathology

## Abstract

**Aims:**

Glioneuronal tumours (GNTs) are poorly distinguished by their histology and lack robust diagnostic indicators. Previously, we showed that common GNTs comprise two molecularly distinct groups, correlating poorly with histology. To refine diagnosis, we constructed a methylation‐based model for GNT classification, subsequently evaluating standards for molecular stratification by methylation, histology and radiology.

**Methods:**

We comprehensively analysed methylation, radiology and histology for 83 GNT samples: a training cohort of 49, previously classified into molecularly defined groups by genomic profiles, plus a validation cohort of 34. We identified histological and radiological correlates to molecular classification and constructed a methylation‐based support vector machine (SVM) model for prediction. Subsequently, we contrasted methylation, radiological and histological classifications in validation GNTs.

**Results:**

By methylation clustering, all training and 23/34 validation GNTs segregated into two groups, the remaining 11 clustering alongside control cortex. Histological review identified prominent astrocytic/oligodendrocyte‐like components, dysplastic neurons and a specific glioneuronal element as discriminators between groups. However, these were present in only a subset of tumours. Radiological review identified location, margin definition, enhancement and T2 FLAIR‐rim sign as discriminators. When validation GNTs were classified by SVM, 22/23 classified correctly, comparing favourably against histology and radiology that resolved 17/22 and 15/21, respectively, where data were available for comparison.

**Conclusions:**

Diagnostic criteria inadequately reflect glioneuronal tumour biology, leaving a proportion unresolvable. In the largest cohort of molecularly defined glioneuronal tumours, we develop molecular, histological and radiological approaches for biologically meaningful classification and demonstrate almost all cases are resolvable, emphasising the importance of an integrated diagnostic approach.

## Graphical abstract

Histological classification of glioneuronal tumours inadequately reflects their underlying biology, and characteristic *BRAF/FGFR1* variants are not always detected. As such, a proportion of cases remain unsolvable. In a large cohort of molecularly classified glioneuronal tumours, we constructed a methylation‐based classification model and identified histological and radiological features that correlate with molecular subtype. We demonstrate that combined methylation, histological and radiological classification can resolve almost all glioneuronal tumours, highlighting the importance of an integrated diagnostic approach for these tumours.

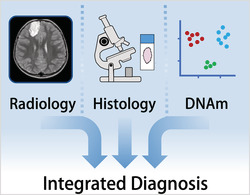

**Key Points**

- Histological classification of glioneuronal tumours inadequately reflects their underlying biology, and characteristic *BRAF/FGFR1* variants are not always detected. As such, a proportion of tumours remain unsolvable.
- In a large cohort of glioneuronal tumours, classified into two groups by molecular profiling, we constructed a methylation‐based classification model that demonstrates high fidelity for glioneuronal tumour molecular classification and performs favourably against histological classification.
- Through comprehensive histological and radiological reviews, we identified features that meaningfully correlate with molecular subtype and demonstrated that combined methylation, histological and radiological analyses can resolve almost all glioneuronal tumours.
- Our study refines the standards for common glioneuronal tumour classification, highlights the importance of an integrated diagnostic approach for accurate stratification and indicates the need for diagnostic terms and criteria that reflect molecularly defined subtypes.

## INTRODUCTION

Glioneuronal tumours (GNTs) are the commonest epilepsy‐associated tumours arising in young children [1]. They present a significant diagnostic problem due to a lack of consistent discriminatory histological features. Poor inter‐observer correlation is common, particularly for the two recognised subtypes comprising the majority of glioneuronal tumours: ganglioglioma (GG) and dysembryoplastic neuroepithelial tumour (DNET). In addition, many cases present with uninformative or mixed histology that inhibits accurate segregation into either entity. Illustrating this point, there is extensive geographical variability across surgical series that cannot be explained by demographic factors, suggesting differences in diagnostic practice and subjective interpretation of histology (reviewed in Thom et al. [2]). This diagnostic problem also extends to the radiological presentation of glioneuronal tumours. They can structurally mimic—or be associated with—focal cortical dysplasia and possess overlapping radiological features [3].

Molecular data are increasingly important for the classification and diagnosis of CNS tumours. An example is the Molecular Neuropathology Platform (MNP) CNS tumour classifier, which compares the methylation profile of a sample against a reference dataset to recommend a classification [4]. However, in practice, these tools perform poorly for low‐grade and glioneuronal tumours compared with more well‐defined tumours. In our recent evaluation of the MNP classifier within routine clinical practice, only 28/85 (33%) of cases within the low‐grade glioma and glioneuronal spectrum could be classified confidently [5]. Similarly, in a large multi‐centre cohort of paediatric low‐grade glioma, MNP achieved a high confidence classification in only 44% of cases [6]. Moreover, in a recent cohort of glioneuronal tumours, only 12/46 (26%) could be robustly classified with the same tool [7]. This imprecision may reflect that low‐grade glioneuronal reference cohorts for these systems were segregated and constructed using inconsistent, and sometimes subjective, histological criteria. To address this, molecularly informed systems for classification, based on objective metrics, are necessary to accurately segregate entities based on underlying biology. Subsequently, refined classification reflecting tumour biology has the potential to facilitate improvements in diagnosis, targeted treatment and the development of meaningful trials and therapeutic strategies, all areas where glioneuronal tumours are lacking compared with other CNS tumours.

Previously, we used data from RNA expression sequencing and DNA methylation arrays to show that the underlying molecular profiles of glioneuronal tumours are not adequately captured by histological classification [8]. We showed that rather than categorising by histological criteria into ganglioglioma and DNET, glioneuronal tumours are better split into two molecular groups by expression and methylation profiles. These groups have different mutational profiles and are enriched, although not completely, for *BRAF* (Group 1) or *FGFR1* (Group 2) variants, respectively. The predominant cell‐type expression profiles align with this molecular classification, corresponding to an astrocytic (Group 1) or an oligodendrocytic (Group 2) enrichment. Clinically, molecular classification correlates with age at onset of seizures, represented by significantly earlier onset in patients possessing Group 1 tumours. Lastly, this molecular classification was able to categorise glioneuronal tumours that could not be classified by conventional histopathology, enhancing the number of tumours that could be resolved.

Here, we aimed to develop methods to apply a molecularly informed classification of glioneuronal tumours, independent of histological archetype. Firstly, we used methylation data from our previous cohort to train a methylation‐based support vector machine (SVM) model to predict classification of glioneuronal tumours into two molecularly defined groups, identified previously. Secondly, we identified radiological correlates that align closely with molecular classification to form a toolkit for incorporation into the early diagnostic workflow. Lastly, we reviewed individual histological features of molecularly classified glioneuronal tumours to identify features that sensitively and specifically segregate them. We subsequently validated all data in a novel cohort of glioneuronal tumours, where both methylation and radiological methods display high fidelity. Taken together, we propose these methods can improve classification of glioneuronal tumours in a manner faithful to the underlying biology as part of an integrated diagnostic workflow.

## METHODS

### Cohorts

Training and validation cohorts were drawn from diagnostic archives and the Children's Cancer and Leukaemia Group Tissue Bank. For methylation analysis, the training cohort consisted of cases with IlluminaHumanMethylation450K data that had classified by their methylation profile (*n* = 36) as Group 1 (*n* = 21) and Group 2 (*n* = 15) in our previous cohort [8]. For radiological analysis the training cohort comprised 31 cases that classified as Group 1 (*n* = 15) or Group 2 (*n* = 16) by either methylation profile or RNA sequencing in the same previous cohort. Radiological data at presentation for these 31 cases were reviewed to identify radiological features associating with molecular class. In total the combined methylation/radiology training cohorts consisted of 49 individual cases and partially overlapped, with 18 cases represented in both (Table S1).

To construct a validation cohort all tumours originally diagnosed as DNETs, ganglioglioma or GNT NOS (glioneuronal tumours with non‐specific features) on their clinical reports over a four‐year period were retrieved. Cases were reviewed histologically according to WHO criteria to confirm a glioneuronal tumour diagnosis and exclude the possibility of alternative specific low‐grade glioma diagnoses. Subsequently, cases were selected for inclusion where sufficient formalin‐fixed paraffin‐embedded (FFPE) material was available to facilitate methylation array analysis (*n* = 34). Thirty‐one of these cases were associated with radiological data that enabled validation of identified radiological features.

Four tumours were represented in both the training and validation cohorts (total *n* = 8). These pairs represented material from separate longitudinal operations for the same tumour. Data for these samples are contained within Table S2.

### DNA preparation

DNA was extracted from FFPE tissue using the Maxwell 16 FFPE Tissue LEV DNA Purification Kit on a Maxwell 16 Research Instrument (Promega, USA) according to manufacturer's instructions. Two hundred fifty nanograms of eluted DNA was subjected to bisulphite conversion using the Zymo EZ DNA Methylation‐Gold Kit (Zymo Research, USA). Bisulphite converted DNA was additionally treated using the Infinium FFPE DNA restore Kit prior to assay on the Illumina HumanMethylationEPIC BeadChip platform (Illumina, USA).

### Mutation screening by targeted panel sequencing

After aliquoting DNA for bisulphite conversion, the remainder was used for targeted panel sequencing. DNA was screened against a panel covering genes that are either clinically actionable or recurrently altered in paediatric cancers [9]. Prior to sequencing, DNA was assayed via Qubit 2.0 fluorometer (ThermoFisher, USA) and TapeStation 2200 (Agilent, USA) to determine quantity and degree of fragmentation. Library preparation, sequencing and variant calling were performed as described in George et al. [9]. Additionally, *FGFR1* tyrosine kinase domain duplications were visually confirmed by manual inspection of sequence data in IGV (v2.5.3). For summary mutation data of the validation cohort; see Table S1. Pathogenicity was determined as pathogenic, variant of uncertain significance (VUS), or likely benign based on annotation within NCBI ClinVar and COSMIC databases. Variants with no records in either database were recorded as VUS.

### Methylation array analysis

Methylation arrays were chosen for predictive model construction and molecular analyses of the validation cohort due to their reproducibility, ability to control batch variation via built in control probes and amenability to FFPE material. DNA from the training cohort had previously been assayed on the Illumina HumanMethylation450 BeadChip platform according to manufacturer's instructions [8]. For the validation cohort, bisulphite converted DNA was assayed on the Illumina HumanMethylationEPIC BeadChip platform (Illumina, USA) in the same manner. Bioinformatic analysis of methylation data was performed in *R*. Prior to analysis, raw data were imported into R using *minfi* [10]. Samples where >10% of probes on the array had failed to hybridise (*n* = 3) were flagged as suboptimal but were retained for subsequent analysis, and 450 K and EPIC array data were harmonised to produce a single unified dataset using the *combineArrays* function and normalised with functional normalisation method as implemented by *minfi*. Probes mapping to the X and Y chromosomes were excluded. In addition, probes located within 50 bp of an SNP, probes with at least one cross‐reactive target and probes with a minor allele frequency >5% were excluded [11]. Following quality control, consensus clustering of methylation data was performed using the *ConsensusClusterPlus* package according to the ‘Ward D2’ implementation of Ward's clustering method [12]. For clustering, the top 10,000 most variable probes within the training cohort according to median absolute deviation were used; mirroring our previous experiment [8]. Beta values for these CpGs were extracted from the combined training and validation cohort data. These were used for consensus clustering of the combined cohort. For classification of samples with the MNP classifier, raw data were read into *minfi* before being passed to MNP (version 11b6) for automatic processing [4]. Version 11b6 represents the most recent version of MNP available to us at the time these analyses were performed. MNP classifications were recorded and considered acceptable with a tumour class calibrated score ≥0.5. Classifications below this score were recorded, but not considered robust as indicated by the authors of the tool [13].

### SVM model construction and testing

Methylation data from the training cohort, where cases had previously classified into two molecular groups, were used to construct a support vector machine (SVM) classification model using the *caret* package in R [8, 14]. The top 10,000 most variable probes across the training cohort were selected, as above. This matrix, alongside corresponding grouping labels, were passed to the *train* function implemented by *caret* to construct a classification model. In brief, the data were centred and scaled during pre‐processing. A classification model was constructed using a radial bias function kernel with ‘leave‐one‐out’ cross validation for internal testing. Up‐sampling was performed during model construction to mitigate differences between training class sizes. Tuning parameters Sigma and Cost were trialled iteratively using a grid search for optimal fit, determined by accuracy, before final model construction.

To assess the model, corresponding data for the 10,000 CpG sites within the model were extracted from the methylation profiles of the validation cohort. This matrix was passed to the model for classification and the output was recorded. Results were compared with consensus clustering and variant data to gauge rationality of SVM classification.

### Radiological imaging protocol

All conventional imaging sequences available on the presentation scan were included for analysis. As imaging had been undertaken in different settings, imaging protocol and scanner type could not be standardised. All cases had the following sequences as standard: T1 pre‐ and post‐contrast, T2, T2 FLAIR and DWI‐ADC.

An unblinded imaging review of all 31 cases in the radiology training cohort was performed by two experienced consultant neuroradiologists (K. M. and A. P. T.) to identify features associated with each molecular group. Five features were identified as potential discriminators between groups: margin of lesion, presence and characteristics of contrast enhancement, presence of the FLAIR‐rim sign on T2 FLAIR [15], location of lesion (temporal vs. extra‐temporal), extension to ependymal surface. Each set of scans was evaluated, the results collated and recorded (Table 2).

To validate results of the unblinded analysis, a third consultant neuroradiologist (W. J.) was provided with a summary of the identified radiological discriminators and performed a blinded classification of the same cohort.

### Histological review

Histological review was performed by an experienced paediatric neuropathologist (T. S. J.) as described previously [8]. Descriptive definitions for scoring specific histological features can be found in Table S3. Control samples used in this study were archival temporal cortex from patients that had undergone resections for hippocampal sclerosis. Prior to inclusion, this material was assessed and confirmed to be free from tumour and other structural pathology.

### Statistical analysis

Fisher's exact test was applied to evaluate the association of clinical, radiological and histological features that correlated with molecular group or diagnosis. In all cases, a *p*‐value <0.05 was considered statistically significant.

### Code availability

R code used to pre‐process methylation data, perform clustering, produce t‐SNE plots and train the SVM model is available at https://github.com/tj-stone/svmGNT.

## RESULTS

### Characteristics of the validation cohort

Initial radiological analysis and SVM model construction was performed on our previously reported cohort of glioneuronal tumours [8]. However, to validate any findings, we required a validation cohort. To construct a validation cohort, tumours diagnosed as DNET, ganglioglioma, or GNT NOS over a 4‐year period were retrieved (*n* = 34). In total, we retrieved 14 DNET, 15 GG and 5 GNT NOS. Summary clinical data are shown in Table S4. The cohort was equally split between male and female. Mean ages at diagnosis and surgery were 67.3 and 111.7 months, respectively, and 32/34 (94%) patients presented with seizures at diagnosis. Overall, the mean clinical follow up from last surgery was 24.8 months and across the cohort 24/32 (75%) of patients were seizure free (Engel Class I) at this point. There were no significant associations between histological diagnosis and any of these clinical features.

### Unsupervised consensus clustering reiterates a two‐group dynamic for glioneuronal tumours

**FIGURE 1:**
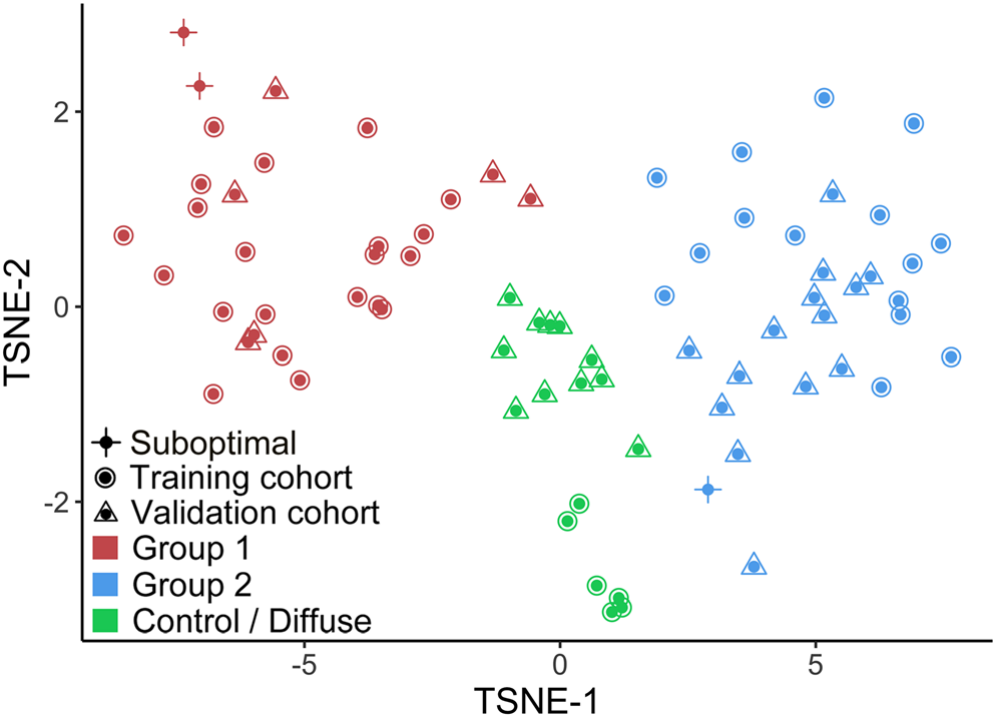
Combined methylation training and validation cohorts cluster into two groups by consensus clustering. tSNE visualisation labelled according to cohort (shape) and consensus classification (colour). Consensus clustering splits the combined cohort into two tumour groups: Group 1 (red) and Group 2 (blue). A small number of cases in the validation cohort cluster alongside temporal lobe controls from the training cohort (green), suggesting low tumour content. Three samples with >10% array probe failure are marked as suboptimal.

Having identified a validation cohort of 34 glioneuronal tumours, our first aim was to perform clustering of the methylation array data to identify outliers and resolve molecular classifications that could be compared against our models. When the validation cohort was introduced alongside the training cohort, we saw no changes in the classifications of our original cohort. Cases from the validation cohort clustered into groups alongside those from the original methylation cohort (Figure 1). In total, 23/34 validation cohort glioneuronal tumours segregated this way. The remaining 11 cases clustered with control temporal lobe tissue. This was likely caused by admixed tumour/non‐tumour cells in these samples resulting in low tumour content and a weak methylation profile. This was an effect we noted previously due to the diffuse nature of some glioneuronal tumours. Taken together, this finding reiterates that the major glioneuronal tumour types belong to two biological groups defined by underlying molecular profiles.

### SVM modelling predicts molecular classification for individual glioneuronal tumours

With our combined cohort, we had been able to perform a consensus clustering analysis to derive molecular classifications for 23 tumours in our validation cohort. However, in clinical practice glioneuronal tumours present as individual cases and a large cohort suitable for clustering and classification is unavailable. To address this and allow individual sample prediction, we constructed a model from the methylation profiles identified in our training cohort. To do this, we used support vector machine (SVM) learning to construct a classification model. We validated the model by assaying the 34 glioneuronal tumours in our validation cohort, including the 23 whose classification had been segregated by consensus clustering. In brief, the model classified 16 tumours as Group 1, 15 tumours as Group 2, and could not distinguish 3 from controls (Table 1; Figure S1).

**TABLE 1:** SVM model predicts tumour classification.

Sample	Histology	BRAF/FGFR1	Consensus clustering	SVM
VAL1	GG	*BRAF* V600E	Group 1	Group 1
VAL2	GG	*BRAF* V600E	Group 1	Group 1
VAL3	GG	‐	Group 1	Group 1
VAL4	GG	‐	Group 1	Group 1
VAL5	GG	‐	Group 1	Group 1
VAL6	GG	‐	Group 1	Group 1
VAL7	GNT NOS	*BRAF* V600E	Group 1	Group 1
VAL8	GNT NOS	‐	Group 1	Group 1
VAL34	DNET	‐	Group 2	Group 1*
VAL16	DNET	*FGFR1‐TACC1*	Group 2	Group 2
VAL17	DNET	*FGFR1* TKD	Group 2	Group 2
VAL18	DNET	*FGFR1* TKD	Group 2	Group 2
VAL19	DNET	*FGFR1* TKD	Group 2	Group 2
VAL20	DNET	*FGFR1* TKD	Group 2	Group 2
VAL21	DNET	*FGFR1* TKD	Group 2	Group 2
VAL22	DNET	*FGFR1* TKD	Group 2	Group 2
VAL23	DNET	*FGFR1* TKD	Group 2	Group 2
VAL24	DNET	*FGFR1* TKD	Group 2	Group 2
VAL25	DNET	*FGFR1* TKD	Group 2	Group 2
VAL26	DNET	‐	Group 2	Group 2
VAL27	DNET	‐	Group 2	Group 2
VAL28	GNT NOS	‐	Group 2	Group 2
VAL29	GNT NOS	‐	Group 2	Group 2
VAL10	GG	*BRAF* V600E	Control/Diffuse	Group 1
VAL11	GG	*BRAF* V600E	Control/Diffuse	Group 1
VAL12	GG	*BRAF* V600E	Control/Diffuse	Group 1
VAL13	GG	*BRAF* V600E	Control/Diffuse	Group 1
VAL32	GNT NOS	‐	Control/Diffuse	Group 1
VAL33	GG	‐	Control/Diffuse	Group 1
VAL9	GG	*BRAF* V600E	Control/Diffuse	Group 1
VAL15	GG	*BRAF* V600E	Control/Diffuse	Group 2
VAL14	GG	*BRAF* V600E	Control/Diffuse	Control
VAL30	DNET	*FGFR1* TKD	Control/Diffuse	Control
VAL31	GG	‐	Control/Diffuse	Control

*Note*: Twenty‐three tumours belonging to the validation cohort clustered alongside the training cohort by consensus clustering. The support vector model (SVM) predicted concordant classification in 22/23 (96%).

* A single misclassification in a sample with >10% array probe failure.

To estimate the accuracy of this classification, we compared the SVM classification against the results of consensus clustering. In 22/23 (96%), the model predicted the correct classification. The single misclassification was a DNET that clustered alongside Group 2 but was called Group 1 by SVM. However, this sample had also been flagged as suboptimal, with >10% methylation array probes failing to hybridise. Thus, the data suggest the model delivers a correct classification with high fidelity when the DNA has hybridised well to the array.

We also assayed our model against eight tumours with weak methylation data that clustered alongside controls by consensus clustering, but where we had detected *BRAF*/*FGFR1* variants by targeted sequencing (seven GG and one DNET). The identified variants were used to estimate the correct molecular class for each tumour. Specifically, we previously showed *BRAF* V600E and *FGFR1* mutations are mutually exclusive and align strongly with Group 1 (*BRAF*) and Group 2 (*FGFR1*) [8]. Interestingly, in five cases with *BRAF* V600E variants, the model concordantly predicted a Group 1 classification where consensus clustering had failed to, suggesting the model may be able to segregate cases with low‐tumour content that typically cluster alongside control brain. In the remaining three, no concordant classification was seen.

Taken together, these data indicate the model faithfully reproduces molecular classification, calling almost all cases with consensus classifications concordantly (96%). Additionally, the model showed proficiency in classifying select cases with weak methylation profiles.

### Radiological features correlate with molecular classification

Having developed a working model for glioneuronal tumour classification from methylation array data, we aimed to investigate whether radiological data could factor into a combined diagnostic toolkit. Specifically, we aimed to identify radiological features that are associated with the molecular class. These could feed into an integrated diagnosis, either adding more evidence towards a diagnosis when integrated with other data streams, or potentially informing classification prior to surgery at a time point where molecular and histopathological methods have not been applied.

**FIGURE 2:**
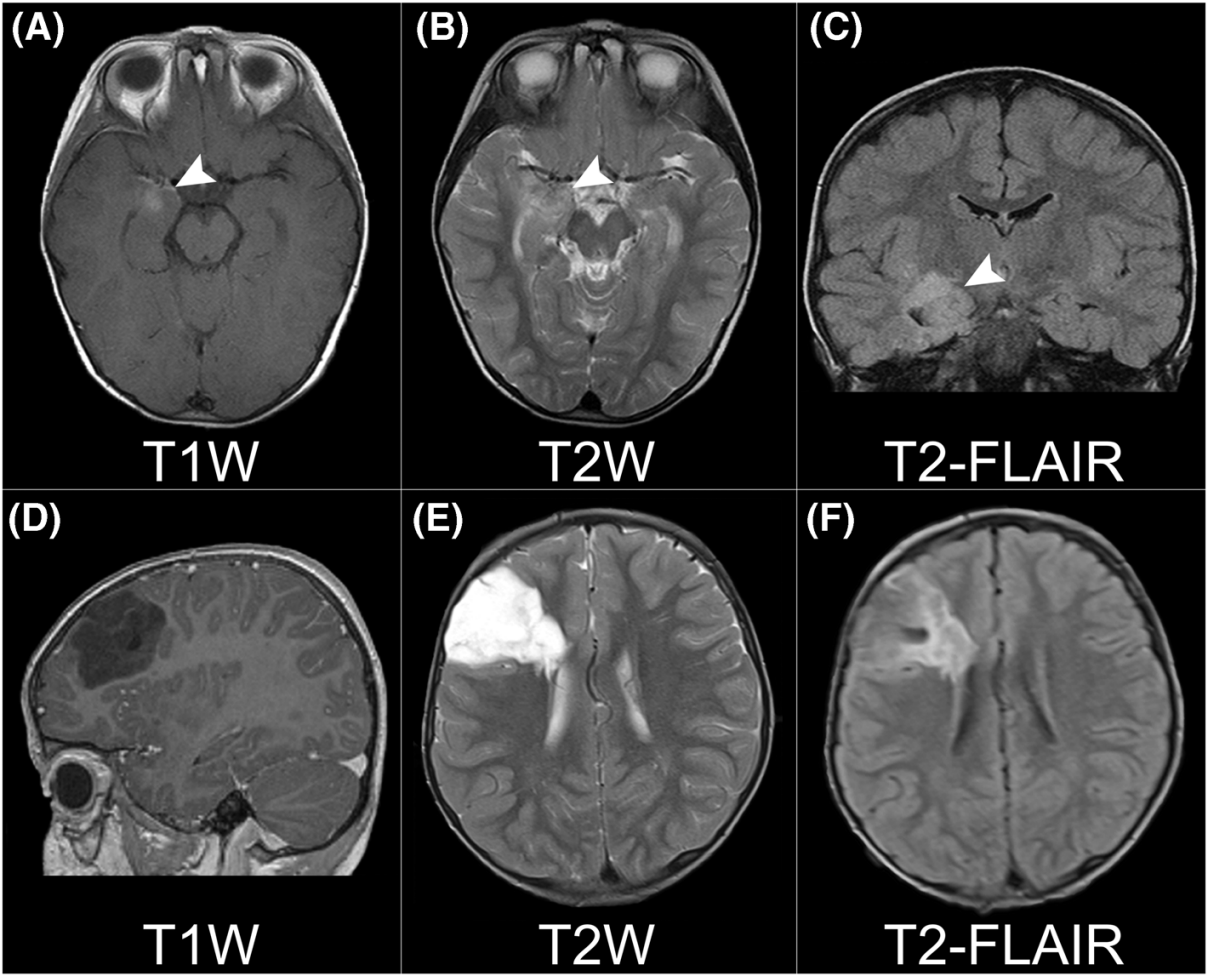
T1 post‐contrast, T2 and T2 FLAIR sequences for example Group 1 (A–C) and Group 2 (D–F) tumours. The Group 1 tumour displays ill‐defined (out‐of‐focus) contrast enhancement (A), is visible with poorly marginated high T2 signal (B) and is located within the medial temporal lobe (A–C). The Group 2 tumour is circumscribed on T1 (D) and T2 (E) sequences, displaying well marginated high T2 signal in the latter. The Group 2 tumour is extra‐temporal and possesses an extension towards the ventricular surface (E) plus a characteristic T2 FLAIR‐rim sign (F), seen as a well‐defined rim of hyperintense signal around the tumour.

From our previous cohort of glioneuronal tumours, we retrieved 31 tumours (15 Group 2, 16 Group 2) and reviewed associated radiology. Summary data for the imaging characteristics of these tumours are presented in Table 2. We observed several features that discriminated strikingly between Group 1 and Group 2 glioneuronal tumours (Figure 2). Group 1 tumours were characterised by ill‐defined tumour margins (15/15), location within the medial temporal lobe (12/15), and ill‐defined or patchy (out‐of‐focus) contrast enhancement (11/15). In contrast, Group 2 tumours were differentiated by circumscribed margins (16/16), presence of T2 FLAIR‐rim (14/16), extra‐temporal location (12/16), and a tail‐like extension to the ventricles (11/16). All listed factors were statistically significant.

**TABLE 2:** Radiological correlates with molecular classification in the radiology training cohort.

	Group 1	Group 2	*p*
Circumscribed margin	0/15 (0%)	16/16 (100%)	<0.005
CE absent	4/15 (27%)	8/16 (50%)	0.273
Well‐defined CE	0/15 (0%)	8/16 (50%)	<0.005
Patchy CE	11/15 (73%)	0/16 (0%)	<0.005
FLAIR rim	0/15 (0%)	14/16 (88%)	<0.005
Temporal location	12/15 (80%)	4/16 (25%)	<0.005
Ventricular tail	0/15 (0%)	11/16 (69%)	<0.005

*Note*: Ill‐defined margins, temporal location and ill‐defined or patchy (out‐of‐focus) contrast enhancement are the strongest Group 1 correlates. Circumscribed margins, T2 FLAIR‐rim, extra‐temporal location, and a tail‐like ventricular extension associate with Group 2.

Abbreviation: CE, contrast enhancement.

To test the reproducibility of radiological features, we recruited a third neuroradiologist who was blinded to tumour classification. Assessing only by the features listed above, 27/31 (87%) of tumours in the radiology training cohort were classified in concordance with the correct molecular classification. Taken together, the high rate of agreement between unblinded and blinded reviewers indicates high fidelity for the identified features.

### Radiological features can prospectively classify glioneuronal tumours

Having identified features that were strongly associated with molecular class in our radiology training cohort we tested these in our validation cohort, where 31 cases had corresponding radiological data. Segregated by radiology, the cohort was classified into 11 Group 1 and 20 Group 2 tumours (Table 3). Mirroring the training cohort, the most frequent features in predicted Group 1 tumours were ill‐defined margins (10/11) and temporal location (10/11). However, the third previously identified feature, ill‐defined or patchy (out‐of‐focus) contrast enhancement, could only be identified in a minority of these cases (4/11). In contrast, all radiological features that were associated with Group 2 tumours in the training cohort were seen in the majority of predicted Group 2 validation tumours. The most frequent of these were a well‐defined or circumscribed margin (19/20), T2 FLAIR‐rim sign (17/20), and a tail‐like extension to the ventricle (16/20) (Table S1).

**TABLE 3:** Radiological classification of validation cohort glioneuronal tumours.

Sample	Histology	BRAF/FGFR1	Consensus clustering	Radiology
VAL1	GG	*BRAF* V600E	Group 1	Group 1
VAL5	GG	‐	Group 1	Group 1
VAL7	GNT NOS	*BRAF* V600E	Group 1	Group 1
VAL2	GG	*BRAF* V600E	Group 1	Group 2*
VAL3	GG	‐	Group 1	Group 2*
VAL4	GG	‐	Group 1	Group 2*
VAL6	GG	‐	Group 1	Group 2*
VAL8	GNT NOS	‐	Group 1	Group 2*
VAL16	DNET	*FGFR1‐TACC1*	Group 2	Group 1*
VAL17	DNET	*FGFR1* TKD	Group 2	Group 2
VAL18	DNET	*FGFR1* TKD	Group 2	Group 2
VAL19	DNET	*FGFR1* TKD	Group 2	Group 2
VAL20	DNET	*FGFR1* TKD	Group 2	Group 2
VAL21	DNET	*FGFR1* TKD	Group 2	Group 2
VAL22	DNET	*FGFR1* TKD	Group 2	Group 2
VAL23	DNET	*FGFR1* TKD	Group 2	Group 2
VAL24	DNET	*FGFR1* TKD	Group 2	Group 2
VAL26	DNET	‐	Group 2	Group 2
VAL27	DNET	‐	Group 2	Group 2
VAL28	GNT NOS	‐	Group 2	Group 2
VAL29	GNT NOS	‐	Group 2	Group 2
VAL34	DNET	‐	Group 2	Group 2
VAL10	GG	*BRAF* V600E	Control/Diffuse	Group 1
VAL12	GG	*BRAF* V600E	Control/Diffuse	Group 1
VAL13	GG	*BRAF* V600E	Control/Diffuse	Group 1
VAL14	GG	*BRAF* V600E	Control/Diffuse	Group 1
VAL15	GG	*BRAF* V600E	Control/Diffuse	Group 1
VAL31	GG	‐	Control/Diffuse	Group 1
VAL9	GG	*BRAF* V600E	Control/Diffuse	Group 1
VAL32	GNT NOS	‐	Control/Diffuse	Group 2
VAL33	GG	‐	Control/Diffuse	Group 2

*Note*: Twenty‐eight tumours could be assessed by radiology alongside consensus clustering or *BRAF/FGFR1* data for comparison. Twenty‐two (78%) classified concordantly. Misclassifications are marked by *.

As with our SVM model, to estimate the fidelity of radiological predictions, we compared these against consensus clustering classification and variant data. Twenty‐eight validation cohort tumours that had been classified by radiology possessed a consensus clustering classification or detectable *BRAF*/*FGFR1* variant. Of these, 22 (78%) radiological classifications were concordant with the molecular findings. The remaining six cases were classified discordantly as Group 1 (*n* = 1) or Group 2 (*n* = 5) tumours by radiology. Of those misclassified as Group 2, all had radiological features associated with Group 2 and lacked consistent Group 1 identifiers. All five had circumscribed margins; three displayed a T2 FLAIR‐rim sign, and three possessed a tail‐like extension to the ventricle. The single case misclassified as Group 1 had ill‐defined margins and a temporal location, associated with Group 1. However, there were no other Group 1 or Group 2 associated features present, suggesting this case lacked specific indicators.

In most cases radiological classification successfully identified the correct classification, suggesting a positive role for radiological classification in early diagnosis, particularly pre‐operatively when other diagnostic data may be limited. However, this approach should be used with caution in instances with limited radiological data. Our analysis shows a small potential for misclassification, skewed towards Group 2. This seems to be led by features with overlap between groups (e.g., location) or those that are interpreted more subjectively (e.g., the circumscription of margins). This could be partially mitigated by favouring more specific features, such as a T2 FLAIR‐rim or ventricular tail, which occurred less frequently in tumours discordantly called Group 2. Additionally, although radiology may not be completely reliable for early classification in all cases, radiological features may serve as useful indicators to form an integrated classification alongside other data later in the clinical timeline, such as molecular or variant data.

### Individual histological features do not robustly replicate molecular classification

Glioneuronal tumour classification by conventional histopathological techniques is impaired by uninformative histology, interpreted subjectively, with poor inter‐observer agreement (reviewed in Thom et al. [2]). Having investigated two systems for predicting glioneuronal tumour classification, we aimed to compare these against histology. To facilitate this, we first reviewed the histological features in our combined training cohorts, where sufficient tissue was available for a detailed analysis (*n* = 46/49), to identify those significantly associated with the molecular class (Table 4; Figure S2). Descriptive definitions for all features assessed can be found in Table S3.

**TABLE 4:** Histological features versus molecular classification in the training cohort.

	Group 1	Group 2	*p*
Astrocytic component	23/27 (85%)	8/19 (42%)	<0.005
Dysplastic neurons	22/27 (81%)	4/19 (21%)	<0.005
Inflammation	15/27 (55%)	2/19 (11%)	<0.005
EGB	13/27 (48%)	1/19 (5%)	<0.005
Oligodendrocyte‐like cells	9/27 (33%)	17/19 (89%)	<0.005
Glioneuronal element	1/27 (4%)	10/19 (53%)	<0.005
Floating neurons	2/27 (7%)	6/19 (32%)	0.051
Microvascular proliferation	2/27 (7%)	3/19 (16%)	0.635
Calcification	14/27 (52%)	7/19 (37%)	0.377
Anaplasia	3/27 (11%)	1/19 (5%)	0.632
Rosenthal fibres	4/27 (15%)	2/19 (11%)	1

*Note*: A prominent astrocytic component, dysplastic neurons, inflammation, and eosinophilic granular bodies (EGB) associate with Group 1. Oligodendrocyte‐like cells, a specific glioneuronal element, and floating neurons associate with Group 2.

We found that although individual histological features correlated with molecular classification, they did not entirely replicate it (Table 4). Additionally, some features were represented in both groups, limiting discriminative utility. Features associated with Group 1 (*n* = 27) were the presence of a prominent astrocytic population (23/27), dysplastic neurons (22/27), inflammation (15/27) and eosinophilic granular bodies (13/27). Features associated with Group 2 (*n* = 19) were the presence of a prominent oligodendrocyte‐like cell population (17/19), a specific glioneuronal element (10/19) and floating neurons (6/19). The strongest correlates for each group—oligodendrocytic/astrocytic prominence—lacked specificity and each was present in the opposing group to a moderate extent. Thus, the discriminative utility of these is limited. For more specific features, such as the presence of dysplastic neurons or a specific glioneuronal element, their occurrence was limited to only a subset of tumours. Dysplastic neurons were only present in 81% of Group 1 cases, whereas a specific glioneuronal element was only seen in 53% of Group 2 tumours. Taken together, these data highlight the difficulty in classifying glioneuronal tumours by conventional histology. Broader features lack specificity and are seen across groups, whereas more specific features are restricted to only a subset of cases. Additionally, the presence of some features in both groups suggests that histology is only partially reflective of the underlying molecular profiles.

### SVM model classification shows greater sensitivity than histological classification

Having identified the strongest histological correlates to molecular classification in our training cohort, we contrasted these against consensus clustering, SVM and radiological predictions in our validation cohort. To predict classification by histological features, we used the presence of the top 2 histological correlates for each group. This combination was chosen to mediate sensitive and specific features against each other. For Group 1 these features were a prominent astrocytic component and dysplastic neurons. For Group 2, a prominent oligodendrocyte‐like component and specific glioneuronal element were required.

**FIGURE 3:**
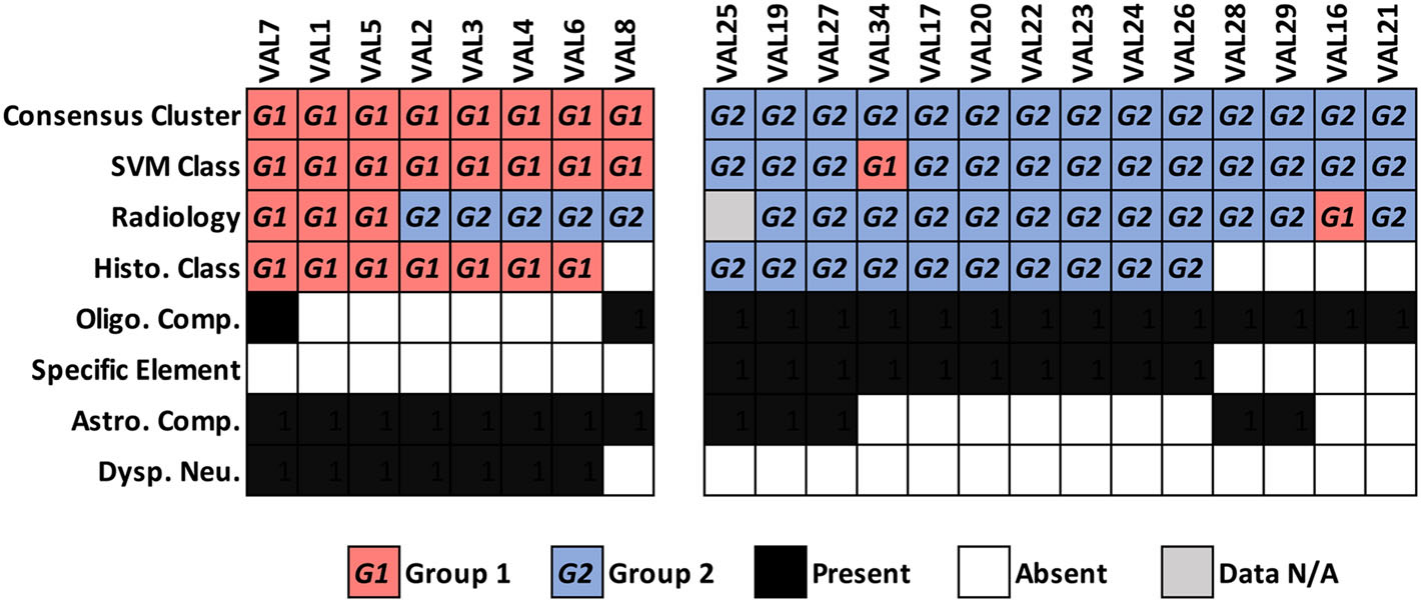
Comparison of histological, SVM, and radiological classifications. Twenty‐two tumours possessed methylation and histological data. Twenty‐one of these also possessed radiological data. SVM and histology perform near equally for Group 1 tumours. However, in Group 2 tumours, histology lacks sensitivity compared to SVM. Radiology predicts most Group 2 tumours but may be biased towards this outcome as indicated by misclassification of Group 1 tumours.

Twenty‐two tumours within the validation cohort had both sufficient material for histological review and methylation data sufficient for classification by consensus clustering into one of the two molecularly defined groups. This sub‐cohort contained 8 Group 1 and 14 Group 2 tumours as defined by consensus clustering (Figure 3). Consensus classification was utilised as a ‘ground truth’ in this comparison, against which other classifications were contrasted. As we had only included cases with sufficient histological material for a detailed analysis, to realise a fair comparison, we excluded cases with weak methylation profiles. These cases were those that clustered alongside controls during the initial consensus clustering analysis, an occurrence that frequently coincides with sparse tumour content in our previous experience. Of the 22 cases with histological and methylation data, 21 possessed assessable radiological data.

Interestingly, we found that histological classification and SVM performed almost equally for Group 1 tumours. SVM predicted all Group 1 tumours correctly, whereas histology failed to classify one tumour, which possessed mixed astrocytic/oligodendrocytic features and lacked dysplastic neurons or a specific glioneuronal element. Conversely, radiological classification performed poorly for Group 1 tumours in this comparative analysis, only concordantly calling 3/8 (37%) of tumours, with the remainder discordantly called as Group 2. Of the five discordant classifications, all possessed circumscribed margins, three possessed tail‐like extensions to the ventricles, and three displayed a T2 FLAIR‐rim. These features were strong Group 2 predictors in the training cohort.

In contrast to Group 1, we observed a greater reduction in sensitivity for histological classification versus SVM when identifying Group 2 tumours. Histological classification concordantly identified 10/14 (71%) Group 2 tumours, compared with 13/14 (93%) for SVM. This is partially explained by the lack of specific histological features. Although all Group 2 tumours contained a prominent oligodendrocytic component, four cases lacked a specific glioneuronal element to confirm histological classification. Moreover, two of these cases had mixed histological features and possessed a prominent astrocytic component. The single misclassification by SVM corresponded to a tumour with >10% methylation array probe failure that had been flagged as suboptimal. Turning to radiology, 13/14 cases had radiological data. A concordant classification was reached for 12 (92%). However, taken together with the misclassification of most Group 1 tumours above, this high calling rate may represent a bias towards Group 2.

Overall, analysis of these methods indicates comparable performance for prediction by histology and SVM in Group 1 tumours, with a marked increase in relative performance for SVM in Group 2 tumours. This emphasises the utility of methylation array classification, where optimal data are available, compared with conventional histology for glioneuronal tumours. Conversely, at least in this cohort, the radiological prediction should be approached conservatively as the criteria for classification may bias towards a Group 2 outcome.

### SVM model classification synergises with existing methylation‐based tools for classification

Methylation profiling to predict tumour diagnosis has expanded alongside the development of tools to analyse and interpret these data. The most notable example for CNS tumours is the Molecular Neuropathology Platform (MNP), a machine learning trained tool based upon random forest classification (https://www.molecularneuropathology.org/mnp) [4, 13]. Previously, we noted this platform performed poorly for low‐grade and glioneuronal tumours, assigning robust classifications to only 33% [5]. This lack of confidence for low‐grade glioma and glioneuronal tumours is aligned with the experiences from other centres [6, 7]. Having compared our SVM model to conventional histopathology, we aimed to assess its competence against this alternative tool, which differs from our model in both underlying classification algorithm and case inclusion criteria. For this, we recorded the classification produced by MNP and whether the calibrated score (a measure of confidence) was ≥0.5, the threshold below which the tool's authors discard classifications [13]. MNP gives classifications according to histological paradigm; thus, we interpreted a GG classification as equivalent to Group 1; likewise, DNET for Group 2 (Table 5).

**TABLE 5:** MNP and SVM model display contrasting performance for classification of glioneuronal tumours.

Sample	Histology	BRAF/FGFR1	Consensus clustering	SVM	MNP
VAL1	GG	*BRAF* V600E	Group 1	Group 1	CONTR, REACT*
VAL2	GG	*BRAF* V600E	Group 1	Group 1	CONTR, REACT*
VAL3	GG	‐	Group 1	Group 1	CONTR, REACT*
VAL4	GG	‐	Group 1	Group 1	PLEX, PED B*
VAL5	GG	‐	Group 1	Group 1	MNG*
VAL6	GG	‐	Group 1	Group 1	PLEX, PED B*
VAL7	GNT NOS	*BRAF* V600E	Group 1	Group 1	LGG, PA/GG ST
VAL8	GNT NOS	‐	Group 1	Group 1	LGG, PA/GG ST
VAL34	DNET	‐	Group 2	Group 1	MB, G3*
VAL16	DNET	*FGFR1‐TACC1*	Group 2	Group 2	LGG, DNT
VAL17	DNET	*FGFR1* TKD	Group 2	Group 2	LGG, DNT*
VAL18	DNET	*FGFR1* TKD	Group 2	Group 2	LGG, DNT
VAL19	DNET	*FGFR1* TKD	Group 2	Group 2	LGG, DNT
VAL20	DNET	*FGFR1* TKD	Group 2	Group 2	LGG, DNT
VAL21	DNET	*FGFR1* TKD	Group 2	Group 2	LGG, DNT
VAL22	DNET	*FGFR1* TKD	Group 2	Group 2	LGG, DNT
VAL23	DNET	*FGFR1* TKD	Group 2	Group 2	LGG, DNT
VAL24	DNET	*FGFR1* TKD	Group 2	Group 2	LGG, DNT
VAL25	DNET	*FGFR1* TKD	Group 2	Group 2	LGG, DNT
VAL26	DNET	‐	Group 2	Group 2	LGG, DNT
VAL27	DNET	‐	Group 2	Group 2	LGG, DNT*
VAL28	GNT NOS	‐	Group 2	Group 2	LGG, DNT*
VAL29	GNT NOS	‐	Group 2	Group 2	LGG, DNT
VAL10	GG	*BRAF* V600E	Control/Diffuse	Group 1	LGG, GG
VAL11	GG	*BRAF* V600E	Control/Diffuse	Group 1	LGG, GG*
VAL12	GG	*BRAF* V600E	Control/Diffuse	Group 1	LGG, GG
VAL13	GG	*BRAF* V600E	Control/Diffuse	Group 1	LGG, GG*
VAL32	GNT NOS	‐	Control/Diffuse	Group 1	LGG, GG
VAL33	GG	‐	Control/Diffuse	Group 1	LGG, GG
VAL9	GG	*BRAF* V600E	Control/Diffuse	Group 1	LGG, GG
VAL15	GG	*BRAF* V600E	Control/Diffuse	Group 2	LGG, GG
VAL14	GG	*BRAF* V600E	Control/Diffuse	Control	LGG, GG
VAL30	DNET	*FGFR1* TKD	Control/Diffuse	Control	CONTR, HEMI*
VAL31	GG	‐	Control/Diffuse	Control	LGG, GG

*Note*: Of the 23 tumours segregated by consensus clustering, MNP classifies 13/23 (56%) concordantly, rising to 16/23 (69%) when confidence threshold is ignored. MNP performs comparably with SVM for Group 2/DNET, whereas Group 1 sensitivity is lower. Both methods perform comparably for cases with weak methylation data, concordantly classifying 5/8 cases alongside adjuvant variant data. MNP column represents classification as listed in MNP reference.

* Misclassifications and classifications below confidence threshold 0.5.

In 23 validation cohort tumours with methylation data that could be segregated into the two molecularly defined groups by consensus clustering, we found that 13 (56%) were concordantly called GG (~Group 1) or DNET (~Group 2) by MNP. If the calibrated score was ignored this rose to 16 (69%). This compared with 22 (96%) that were correctly called by SVM. MNP performed comparably for DNET/Group 2 but struggled with GG/Group 1 tumours, misclassifying 6/8. When we analysed eight samples with methylation data that did not segregate from controls by consensus clustering but had detectable *BRAF/FGFR1* variants, MNP and SVM also performed comparably. MNP called 5/8 tumours correctly with a calibrated score above the cut‐off, rising to 7/8 if the threshold was ignored. All were samples with a *BRAF* V600E alteration and classified as GG by MNP. This compared to 5/8 that SVM classified as Group 1 in concordance with *BRAF* alteration. Taken together, these findings indicate comparable sensitivity between the two methods for detection and classification of DNET/Group 2 tumours. MNP performs poorly for GG/Group 1 tumours but was able to call cases with weak methylation data. However, SVM was also able to segregate samples with weak methylation profiles in concordance with *BRAF/FGFR1* variant data. Notably, when both approaches are used together, they overlap and almost all samples can be classified correctly. As both rely on methylation array data, once an array is run it should be trivial to perform both for a given sample. Lastly, it should be noted that MNP is not a static entity but rather a versioned tool under continuing development. This analysis represents a snapshot using the latest version available at the time of writing. As such, these comparisons are prone to change as existing entities are refined and novel ones incorporated into MNP.

## DISCUSSION

Glioneuronal tumour classification is confounded by reliance on histological features that are frequently uninformative and prone to subjectivity. In our histological analysis, we identified features correlating with molecular classification but none completely replicating it. The most common features were broad cell types, whereas more specific features like dysplastic neurons or a specific glioneuronal element were absent in a significant proportion. Histological classification suffers from a lack of inter‐observer agreement, including between specialist centres, as indicated by widely variable figures for the reported incidence of the main histologically defined glioneuronal archetypes (reviewed in Thom et al. [2]). Outside these centres, glioneuronal tumours are encountered infrequently and recognition is complicated by lack of experience. Difficulty with accurate and reproducible classification extends to research; cohorts may represent a clinically complex mix of tumours segregated by unreliable criteria that do not reflect the underlying molecular biology, impacting the resolving power of downstream analyses. These problems highlight a lack of objective and reproducible ‘gold standards’ for glioneuronal tumours. Addressing this diagnostic problem with biologically informed metrics has the potential to facilitate better diagnosis and the development of meaningful trials and targeted therapeutic strategies. Likewise, robust classification and biological characterisation represents important groundwork towards understanding the heterogeneous survival and morbidity outlook for glioneuronal tumour patients [16, 17, 18, 19, 20].

Recently, methylation arrays have shown significant utility for molecular segregation of CNS tumours through classification tools [4]. These perform well for high‐grade and molecularly well‐defined tumours but are limited for low‐grade and glioneuronal tumours [6, 7] (reviewed in Pickles et al. [5]). To assess the utility of classifying glioneuronal tumours by methylation profiles, we constructed a support vector machine model. We compared this against histological, radiological, variant and molecular consensus clustering data, in addition to classification via another methylation‐based CNS tumour classifier. Our model demonstrated high classification fidelity (96%) when presented with methylation data of suitable quality. Against histological features, we noted comparable, potentially improved, performance with enhanced classification for many samples. Moreover, the model worked synergistically with existing molecular and methylation‐based tools to resolve classifications for almost all samples analysed. Insights into the molecular biology of CNS tumours have promoted an integrated diagnostic approach, incorporating multiple histo‐molecular data streams. Rather than replacing or replicating existing methods, we view methylation profiling as additive to an integrated diagnosis, contributing confidence and objective resolving power in the absence of absolute standards for glioneuronal tumours.

In addition to our SVM model, we identified radiological features associated with molecular classification. To our knowledge, no detailed radiological study of discriminators between molecularly defined glioneuronal tumours has been undertaken. We noted good inter‐observer agreement between independent reporters when using lesion margins, location, enhancement characteristics, T2 FLAIR‐rim and presence of a tail‐like extension to the ventricles as discriminators. Some features are potentially explicable by the underlying biology. For example, T2 FLAIR‐rim enrichment for Group 2 tumours may reflect predominance of DNET‐like tumours in this group, for which the sign is documented [15]. Additionally, differences in tumour margin definition and enhancement potentially highlight underlying cell type enrichments in each group. Group 1 tumours display an enrichment for astrocytic cell types and present in a diffuse pattern, whereas Group 2 are predominantly oligodendrocytic, nodular and circumscribed.

The performance of radiology suffered slightly in our validation cohort compared with other methods and potentially demonstrates a bias towards Group 2. However, to allow a fair comparison, this cohort subset excluded tumours without a consensus clustering classification and those without detailed histological data. Within the excluded cases were seven where radiology predicted concordantly alongside *BRAF* V600E or *FGFR1* variants (Table 3), demonstrating utility in the absence of other data. Moreover, among all radiologically assessable cases 78% could be classified concordantly alongside the available data. As such, we propose that radiological features represent a worthwhile tool for early classification and can guide onward diagnostic testing, improving efficiency by streamlining the early diagnostic pipeline and downstream test selection. Although we focused on conventional imaging, in future studies, it would be worthwhile interrogating advanced imaging sequences to further explore the differences between molecular groups and establish additional imaging biomarkers.

Taken together, our analysis suggests methylation and radiological classification can add significant utility to integrated diagnostic workflows for glioneuronal tumours. Both utilise data that in many centres are standard practice. Pre‐surgical radiological assessment is standard for CNS tumours and methylation array profiling is increasingly common at major centres [5, 13, 21, 22]. As such, application of these methods frequently involves interrogation of existing data with few additional barriers to entry. Moreover, in the context of recent shifts towards the incorporation of molecular data into WHO criteria for specific diagnoses, we expect that molecular classification for glioneuronal tumours will represent an important step towards correct stratification [23]. Indeed, considering the disconnect between histological paradigm and molecular biology for these tumours, we question the continued utility of DNET and ganglioglioma as diagnostic terms, as opposed to identifiers that more accurately reflect a modern understanding of the tumour biology. As such, we propose these terms are abandoned in favour of molecularly defined subtypes.

## AUTHOR CONTRIBUTIONS

Thomas J. Stone, Kshitij Mankad, Darren Hargrave and Thomas S. Jacques conceived the study. Thomas J. Stone, Kshitij Mankad and Thomas S. Jacques wrote the manuscript. Thomas J. Stone, Jessica C. Pickles, Jane Chalker, Iwona Slodkowska, Emily Pang, Mark Kristiansen, Gaganjit K. Madhan and Leysa Forrest prepared methylation arrays and collected data. Kshitij Mankad, Ai Peng Tan and Wajanat Jan collected and analysed the radiology data. Thomas J. Stone, Thomas S. Jacques, Talisa Mistry, Olumide Ogunbiyi and Saira W. Ahmed prepared and analysed the histology data. Deborah Hughes, Eleni Koutroumanidou and Mike Hubankprepared and analysed DNA panel sequencing data. Maria Gogou and J. Helen Cross collected clinical data. Thomas J. Stone performed bioinformatics and statistical analyses. All authors read and approved the final manuscript.

## CONFLICT OF INTEREST

The authors declare that they have no conflicts of interest.

## ETHICS STATEMENT

Institutional approval for this project was granted by the joint ICH/GOSH R&D department with approval from the local research ethics committee, BRAIN UK, and the CCLG Tissue Bank.

### PEER REVIEW

The peer review history for this article is available at https://publons.com/publon/10.1111/nan.12894.

## Supporting information


**Figure S1.** tSNE visualisation of the methylation training and validation cohorts labelled according to cohort (shape) and SVM classification (colour). Three samples with >10% array probe failure are marked as suboptimal.

**Figure S2.** Representative examples of histological features strongly associated with molecular classification. The presence of a specific glioneuronal element (A) is associated with Group 2. Group 2 tumours are also associated with floating neurons (B)(arrowheads) and oligodendrocyte‐like cell enrichment (B)(arrow). Group 1 tumours were associated with a prominent astrocytic component (C) and dysplastic neurons (D)(arrowheads). Magnification 20x (A, C), 40x (B, D). Scale bars 100 μm (A, C), 200 μm (B, D).

**Table S1.** Combined molecular, histological, and radiological data for all 83 glioneuronal tumour samples in the training and validation cohorts.

**Table S2.** Combined molecular, histological, and radiological data for 4 longitudinally sampled tumour pairs present in both the training and validation cohorts.

**Table S3.** Descriptive definitions for specific histological features.

**Table S4.** Validation cohort clinical characteristic summary data.

## Data Availability

The data that support the findings of this study are available on reasonable request from the corresponding author. R code used to pre‐process methylation data, perform clustering, produce t‐SNE plots and train the SVM model is available at https://github.com/tj-stone/svmGNT.
