# Supplementary figures and images for: DNA methylation‐based classification of glioneuronal tumours synergises with histology and radiology to refine accurate molecular stratification
Source: Neuropathol Appl Neurobiol. 2023 Mar 8;49(2):e12894. doi: 10.1111/nan.12894 (PMC10946721; doi:10.1111/nan.12894)

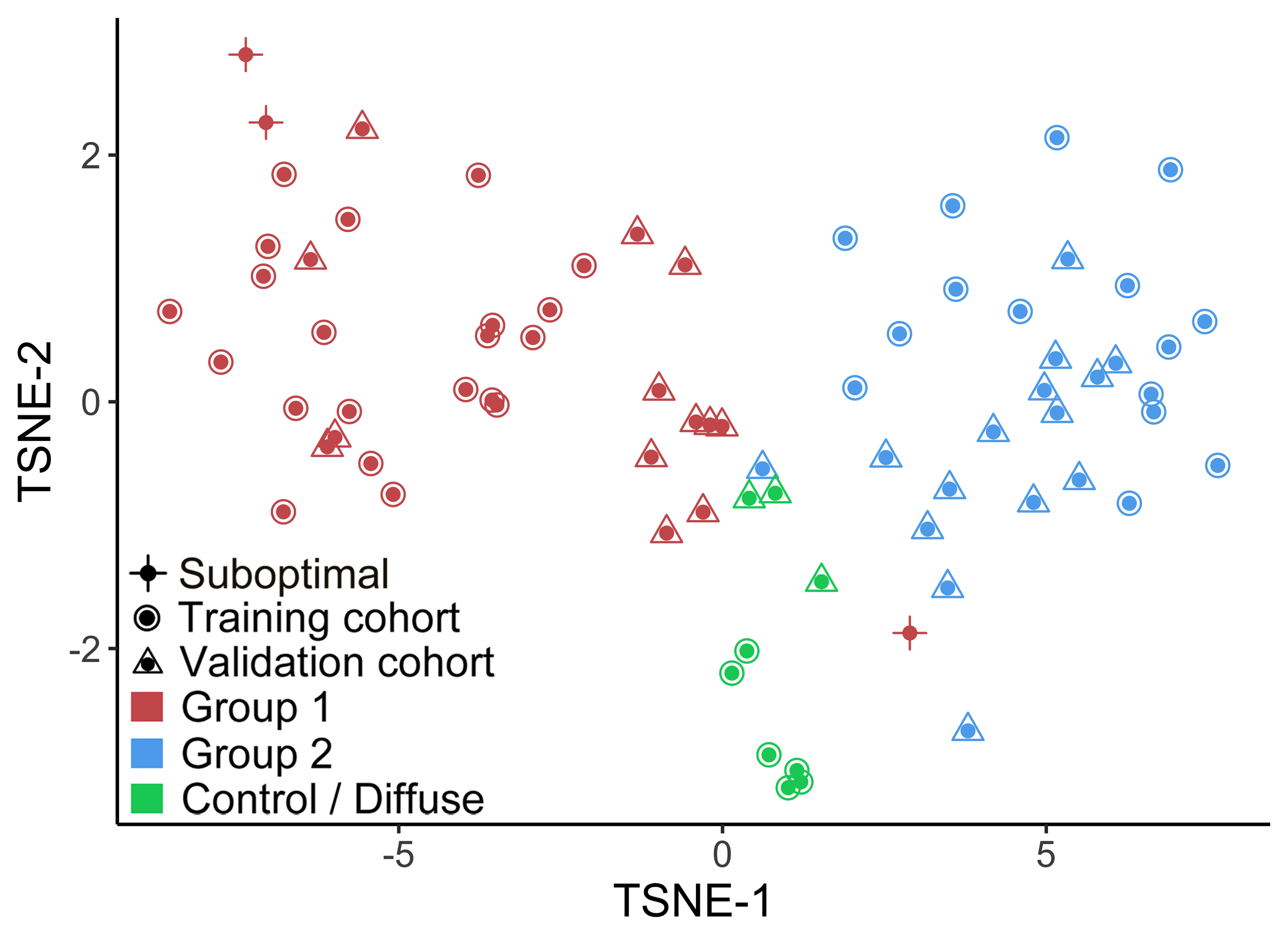

Supplement: Supplementary file 1 — Figure S1. tSNE visualisation of the methylation training and validation cohorts labelled according to cohort (shape) and SVM classification (colour). Three samples with >10% array probe failure are marked as suboptimal. [file NAN-49-0-s001.tif]

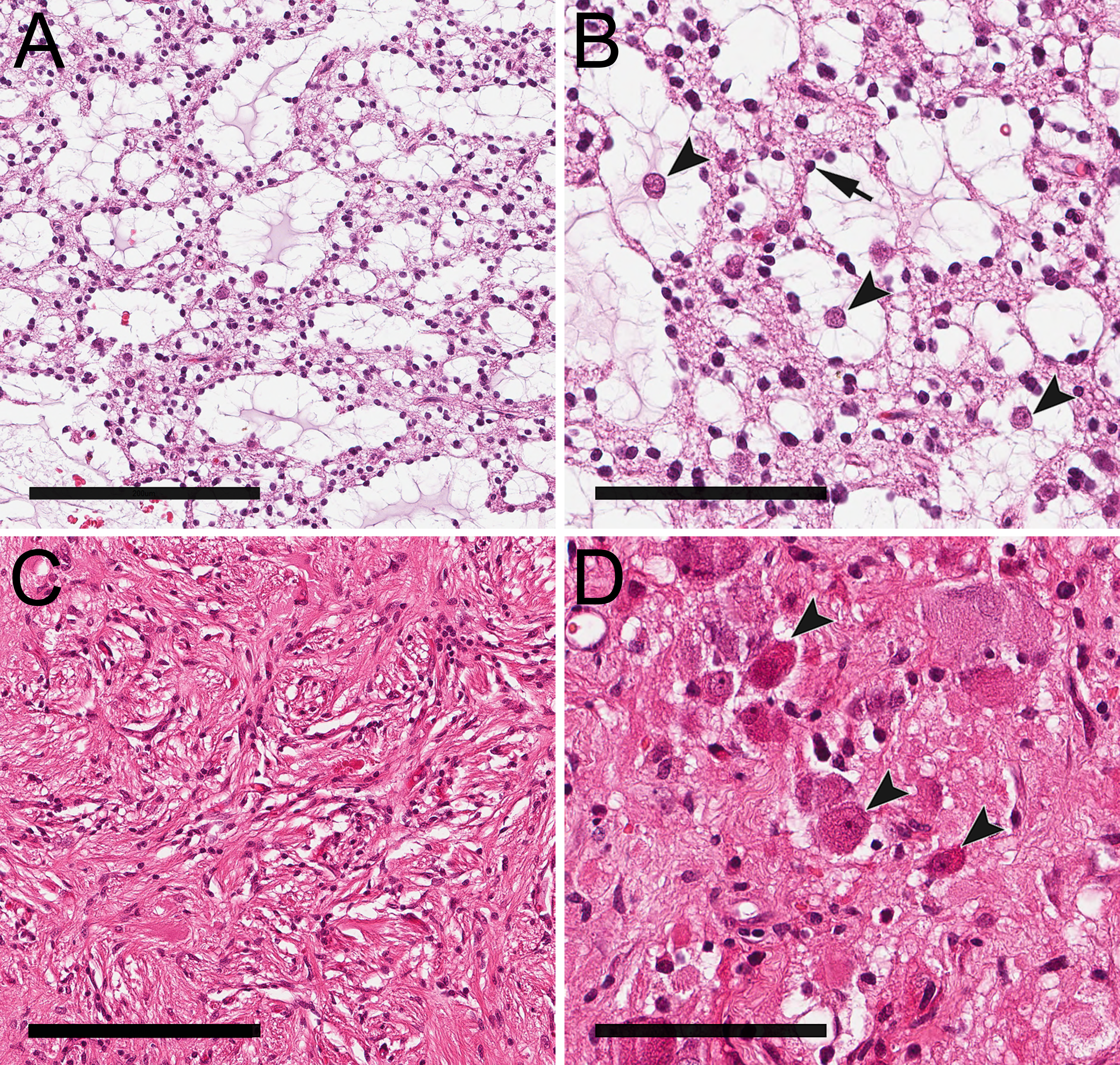

Supplement: Supplementary file 2 — Figure S2. Representative examples of histological features strongly associated with molecular classification. The presence of a specific glioneuronal element (A) is associated with Group 2. Group 2 tumours are also associated with floating neurons (B)(arrowheads) and oligodendrocyte‐like cell enrichment (B)(arrow). Group 1 tumours were associated with a prominent astrocytic component (C) and dysplastic neurons (D)(arrowheads). Magnification 20x (A, C), 40x (B, D). Scale bars 100 μm (A, C), 200 μm (B, D). [file NAN-49-0-s005.tif]
